# Transcriptional Signatures of Immune, Neural, and Endocrine Functions in the Brain and Kidney of Rainbow Trout (*Oncorhynchus mykiss*) in Response to *Aeromonas salmonicida* Infection

**DOI:** 10.3390/ijms23031340

**Published:** 2022-01-25

**Authors:** Mengqun Liu, Xiaodong Yang, Chu Zeng, Hongkui Zhao, Jifang Li, Zhishuai Hou, Haishen Wen

**Affiliations:** Key Laboratory of Mariculture, Ministry of Education (KLMME), Ocean University of China, Qingdao 266003, China; liumengqun0826@126.com (M.L.); yxd19951108@163.com (X.Y.); zengchu1234@126.com (C.Z.); zhaohongkuiouc@163.com (H.Z.); lijf@ouc.edu.cn (J.L.)

**Keywords:** rainbow trout, *Aeromonas salmonicida*, furunculosis, immunomodulation, RNA-Seq

## Abstract

Rainbow trout (*Oncorhynchus mykiss*) serves as one of the most important commercial fish with an annual production of around 800,000 tonnes. However, infectious diseases, such as furunculosis caused by *Aeromonas salmonicida* infection, results in great economic loss in trout culture. The brain and kidney are two important organs associated with “sickness behaviors” and immunomodulation in response to disease. Therefore, we worked with 60 trout and investigated transcriptional responses and enrichment pathways between healthy and infected trout. We observed that furunculosis resulted in the activation of toll-like receptors with neuroinflammation and neural dysfunction in the brain, which might cause the “sickness behaviors” of infected trout including anorexia and lethargy. We also showed the salmonid-specific whole genome duplication contributed to duplicated colony stimulating factor 1 (*csf-1*) paralogs, which play an important role in modulating brain immunomodulation. Enrichment analyses of kidneys showed up-regulated immunomodulation and down-regulated neural functions, suggesting an immune-neural interaction between the brain and kidney. Moreover, the kidney endocrine network was activated in response to *A. salmonicida* infection, further convincing the communications between endocrine and immune systems in regulating internal homeostasis. Our study provided a foundation for pathophysiological responses of the brain and kidney in response to furunculosis and potentially offered a reference for generating disease-resistant trout strains.

## 1. Introduction

Fish serves as an important single source of high-quality protein [1]. Based on the statement of the Food and Agriculture Organization (FAO) of the United Nations, fish consists of around 16% of animal protein consummation worldwide (FAO, 1997; [1]). Due to the global decline of ocean fishery stocks, a *Nature* paper indicated that aquaculture might act as a solution for increased fishery production [2]. For example, aquaculture produced more than 80 million tonnes of fish and shellfish in 2017, providing protein-rich food to consumers from low- to high-income regions (FAO, 2019; [3,4,5,6,7]). Rainbow trout (*Oncorhynchus mykiss*) is one of the most important commercial and market fish with global production around 800 thousand tonnes (FAO, 2020). Compared to dramatically depleted wild fishery resources, cultured fish are making up an increasing proportion of the world’s fishery population [1].

The cultured fish must cope with multiple environmental and pathogen challenges [8,9]. Moreover, the environmental stimuli might result in the stress of fish, thus causing increased susceptibility of disease with pathogen load [10,11]. The bacterium *Aeromonas salmonicida* (*Aeromonas salmonicida*. *A. salmonicida* subsp. *salmonicida*), which has been identified as one of the most infective and lethal pathogens for over 100 years, acts as the causative agent of furunculosis [12,13,14]. Furunculosis derives its name from the development of furuncles (boils) on the skin [15]. After infection, *A. salmonicida* could enter the host cells of skin, gills, and intestines, and then rapidly infect the internal organs, thus resulting in severe symptoms and mortality of the infected individuals [16,17]. The *A. salmonicida* is reported to result in diseases in a wide range of freshwater fish, including salmonids, common carp (*Cyprinus carpio*), European catfish (*Silurus glanis*), *Cottus gobio* and fathead minnow (*Pimephales promelas*), and the seawater species of turbot (*Scophthalmus maximus*), and sea bream (*Sparus aurata*) [18,19]. Fish infected by *A. salmonicida* exhibit high morbidity, severe symptoms, and high mortality, thus resulting in great economic loss [12].

The characteristics, virulence, and genome of *A. salmonicida* has been thoroughly investigated, as well as the symptoms, pathophysiology, and pathology [20,21,22,23,24]. For example, in physiological levels, the fish infected by *A. salmonicida* exhibits the symptoms of anorexia, lethargy, abnormal swimming behavior, and haemorrhagic septicaemia with darkened skin pigmentation, skin ulcers and necrosis, and internal bleeding [20,23,25,26]. On the molecular level, several studies revealed the molecular mechanisms of immunomodulation and inflammation in response to *A. salmonicida* infection, including the expression profiles of cytokines, signaling pathways of toll-like receptors, and activation and migration of immune cells [27,28,29,30,31]. A recent study further confirmed that infection of *A. salmonicida* not only impairs the cell physiology and phagocytosis of trout, but also severely dysregulates gene transcription of immune mediators and disturbs the warning signals related to infection recognition and immunomodulation [32]. In recent decades, the omics analyses provide a big database showing the genes, proteins, and metabolites associated with *A. salmonicida* infection [21,33,34,35,36]. For example, a transcriptome analysis showed an important splenic mciroRNA (miR-155-5p) which is associated with inflammation caused by *A. salmonicida* infection [22]. A recent proteomic analysis revealed kidney HADH (β-hydroxyacyl coenzyme A dehydrogenase) and ALDH9A1 (Aldehyde dehydrogenase family 9 member A1-A) could serve as potential molecular indicators of *A. salmonicida* infection at early stages [37]. Moreover, a recent genome-wide association (GWAS) study investigated the quantitative trait loci of rainbow trout related to *A. salmonicida* resistance [38]. However, most of these studies focus on peripheral tissues rather than the brain. Animal behaviors are mainly regulated by neurotransmitters and neuromodulators of the brain [39]. The infected trout exerted sickness behaviors including anorexia and lethargy, suggesting that furunculosis dysregulated brain functions. The limited information of brain transcriptional signatures in response to *A. salmonicida* infection will limit the better understanding of the immune mechanisms during *A. salmonicida* infection in trout (salmonids).

Although early researchers generally studied the immune and central nervous system (CNS) as separate targets due to the concept of “immune privilege”, the last 30 years has witnessed the birth of brain (CNS)-immune communication and the following significant development [40,41]. Peripheral inflammation causes inflammatory processes in the brain and the activated immune cells (mediators), resulting from infection and inflammation, could directly interact with the brain, thus resulting in brain-regulated sickness behaviors including anorexia and reduced locomotor activity [42,43], which is consistent with furunculosis symptoms in trout. Moreover, previous studies showed that the blood-brain barrier (BBB) is disrupted by bacterial infection in both mammalian and teleost species [44,45], further increasing the possibility that peripheral bacterial infection challenges brain immune homeostasis. Therefore, we investigated whether *A. salmonicida* infection resulted in homogeneously (or heterogeneously) immune effects on CNS and peripheral immune tissues in trout.

In our study, we investigated the transcriptional profiles and functional enrichment of brains (CNS) and kidneys (peripheral tissue) of trout in response to *A. salmonicida* infection. We selected the brain and kidney due to the following reasons: 1. Brain cells exert poor regenerative capacity and dysfunction in the brain (by immune cells and/or modulators) could lead to catastrophic effects on life. However, the immunomodulation status of trout brain in response to pathogen infection has been underestimated, so far. 2. Th kidney is a well-characterized immune tissue in teleost and serves as the major target of bacterial *A. salmonicida* infections [25,36]. Furthermore, salmonid has long been a model for novel gene identification because salmonid-specific whole genome duplication (ssWGD) results in duplications of the functional paralogs [46,47]. Therefore, we further analyzed the sequences, structure, and potential functions of immune genes and their paralogs. Our study may reveal insight into the interaction of CNS and peripheral tissues in responses to furunculosis and provide a foundation for breeding disease-resistant trout strains.

## 2. Results

### 2.1. General Transcriptomic Profiles of the Brain and Kidney in Response to A. salmonicida Infection

**Figure 1:**
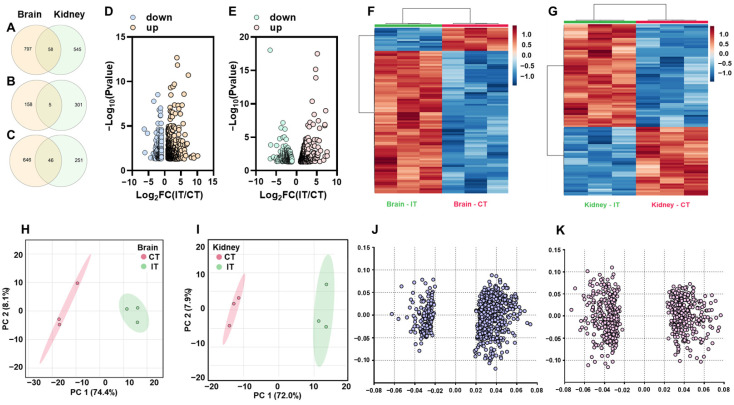
General transcriptomic profiles of trout in response to *A. salmonicida* infection. The differently expressed genes in the brain and kidney in response to *A. salmonicida* infection: (**A**) total (**B**) down-regulation and (**C**) up-regulation. The volcano plots of differently expressed genes in the brain (**D**) and kidney (**E**). The heatmaps of differently expressed genes in brain (**F**) and kidney (**G**). The heatmaps were generated from the counts (normalized by DESeq2) of differently expressed genes. The PCA plots and loading plots of differently expressed genes in the brain (**H**,**J**) and kidney (**I**,**K**). In PCA plots, the red dots show the “vector” of differently expressed genes in CT and green dots shows the “vector” of differently expressed genes in IT. In loading plot, the variables (genes) far from center (0, 0) showed stronger effects on separated clusters in PCA plots.

In trout infected with *A. salmonicida*, RNA-seq analysis showed 855 (163 down and 692 up) and 603 (306 down and 297 up) DEGs in the brain and kidney, respectively (Figure 1A–C). We further observed that 5 DEGs were down-regulated in both brain and kidney, while 46 DEGs showed up-regulation in both the brain and kidney (Figure 1B,C). The statistical results of DEGs are represented in volcano plots and heatmaps (Brain: Figure 1D,F; Kidney: Figure 1E,G). The separated clusters in PCA plots indicated trout in CT and IT exerted different transcriptomic profiles in the brain and kidney (Figure 1H,I). The loading plot showed the values of each gene resulted in separated clusters in PCA plots (Figure 1J,K).

### 2.2. DEGs Commonly Identified in Brain and Kidney

**Figure 2:**
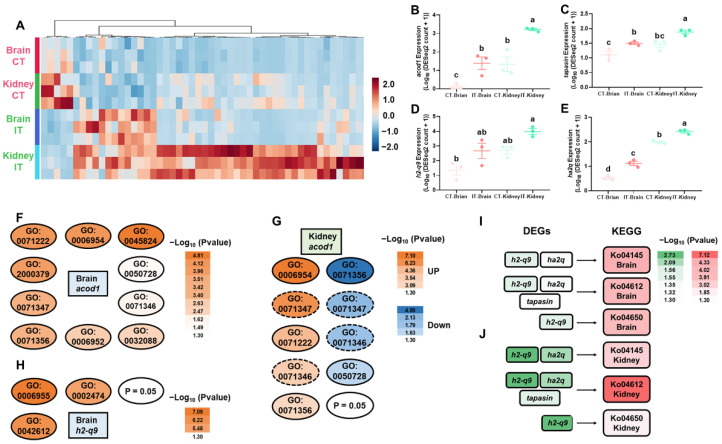
Differently expressed genes commonly identified in the brain and kidney in response to *A. salmonicida* infection. (**A**) The heatmaps of differently expressed genes commonly identified in the brain and kidney; (**B**–**E**) The interleaved scatters of *acod1*, *tapasin*, *h2-q9*, and *ha2q* (Different letters indicate significant differences, *p* < 0.05, one-way ANOVA with Tukey’s test); (**F**,**G**) The GO terms associated with *acod1*. GO:0045824, negative regulation of innate immune response; GO:0006954, inflammatory response; GO:0071222, cellular response to lipopolysaccharide; GO:2000379, positive regulation of reactive oxygen species metabolic process; GO:0071347, cellular response to interleukin-1; GO:0071356, cellular response to tumor necrosis factor; GO:0006952, defense response; GO:0032088, negative regulation of NF-κB transcription factor activity; GO:0071346, cellular response to interferon-gamma; GO:0050728, negative regulation of inflammatory response. (**H**) The GO terms associated with *h2-q9*. GO:0006955, immune response; GO:0042612, MHC class I protein complex; GO:0002474, antigen processing and presentation of peptide antigen via MHC class I. (**I**,**J**) The KEGG pathways commonly enriched in brain and kidney with *h2-q9*, *ha2q* and *tapasin*. Details of heatmaps, GO terms and KEGG pathways are shown in Tables S2–S4.

The common DEGs in both the brain and kidney are shown in heatmaps (Figure 2A). Based on further pathway enrichment analyses, we identified several key genes associated with immunomodulation, including *acod1*, *tapasin*, *h2-q9*, and *ha2q* (Figure 2B–E). The GO analyses showed that *acod1* (with other genes) was involved in inflammatory regulation, cellular defenses, and cellular responses to cytokines in the brain and kidney (Figure 2F,G), and *h2-q9* (with other genes) was associated with immune response and major histocompatibility complex (MHC) functions (Figure 2H). Based on the KEGG database, we observed *h2-q9*, *ha2q*, and *tapasin* (with other genes) were enriched in the pathways of phagosome, antigen processing and presentation, and natural killer cell mediated cytotoxicity (Figure 2I,J).

### 2.3. Enrichment Analysis of DEGs in Brain and Kidney

**Figure 3:**
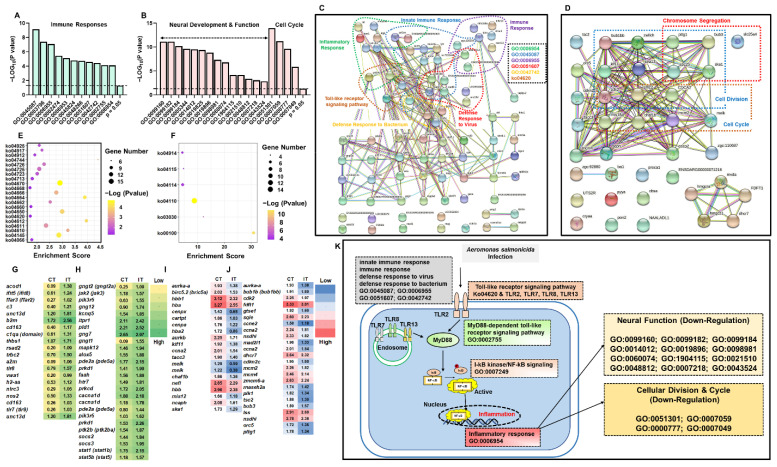
The enrichment analyses of DEGs in the brain in response to *A. salmonicida* infection. (**A**) GO terms associated with immune functions (up-regulated in infected trout); (**B**) GO terms associated with neural functions and cell cycle (down-regulated in infected trout); Predicted functional networks between the up-regulated genes (**C**) and down-regulated genes (**D**). The networks were established by the database of STRING Version 11 (http://string-db.org/, last accessed on 21 December 2021, based on zebrafish). (**E**) KEGG pathways associated with immune and neuro/endocrine functions (up-regulated in infected trout); (**F**) KEGG pathways associated with cell cycle and steroid hormone functions (down-regulated in infected trout). (**G**–**J**) Heatmap of the candidate genes. (**G**) Up-regulated genes enriched in GO terms; (**H**) Up-regulated genes enriched in KEGG pathways; (**I**) Down-regulated genes enriched in GO terms; (**J**) Down-regulated genes enriched in KEGG pathways. (**K**) The putative pathways of trout brain in response to *A. salmonicida* infection. Details of heatmaps, GO terms, and KEGG pathways are shown in Tables S4–S6.

Based on GO enrichment analyses of DEGs, we showed that, in the infected trout, up-regulated DEGs were significantly enriched in GO terms associated with immune functions (Figure 3A), while the down-regulated DEGs were significantly enriched with GO terms of neural functions and cell cycle (Figure 3B). Predicted functional networks between the up-regulated and down-regulated DEGs are shown (Figure 3C,D). The KEGG pathway enrichment analyses indicated that infected trout showed up-regulated KEGG pathways associated with immune and neuro/endocrine functions (Figure 3E, Table S4), while the down-regulated KEGG pathways were involved in cell cycle and steroid hormone functions (Figure 3F, Table S4). The DEGs associated with enriched GO terms and KEGG pathways are shown by heatmap (Figure 3G–J). Based on GO and KEGG analyses, the putative pathways, which were involved in immune defenses, neural functions, and cell cycle of trout brain in response to *A. salmonicida* infection were proposed (Figure 3K, more details in Discussion).

**Figure 4:**
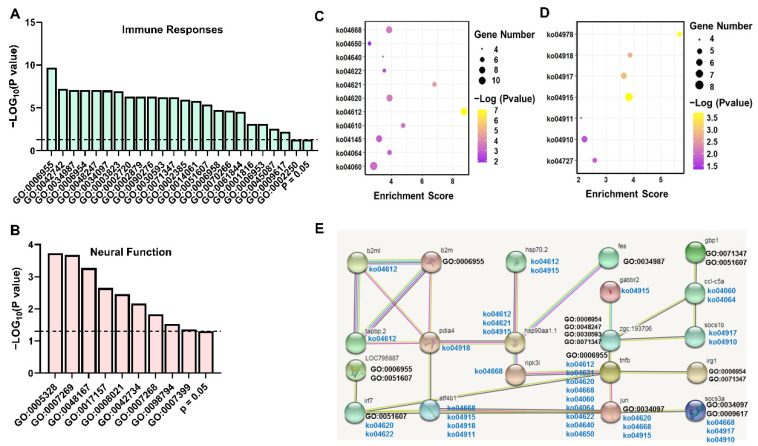
The enrichment analyses of DEGs in the kidney in response to *A. salmonicida* infection. (**A**) GO terms associated with immune functions (up-regulated in infected trout); (**B**) GO terms associated with neural functions (down-regulated in infected trout). (**C**) KEGG pathways associated with immune functions (up-regulated in infected trout); (**D**) KEGG pathways associated endocrine functions (up-regulated in infected trout). (**E**) Predicted functional networks between the DEGs. The networks were established by the database of STRING Version 11 (http://string-db.org/, last accessed on 21 December 2021, based on zebrafish). Details of GO terms and KEGG pathways are shown in Table S4.

The RNA-seq data showed that *A. salmonicida* infections resulted in great influences on immune stimulation, neural functions, and endocrine systems in the kidney, which is consistent with the results in brain. Based on GO enrichment analyses, the infected trout showed up-regulated GO terms involved in immune functions (Figure 4A, Table S4) and down-regulated GO terms associated with neural functions (Figure 4B, Table S4). The KEGG pathway analyses showed the immune-related pathways and endocrine functions were upregulated (Figure 4C,D, Table S4). Functional networks between DEGs were predicted by the database of STRING Version 11 (Figure 4E).

### 2.4. Identification of the Novel Macrophage csf-1r

**Figure 5:**
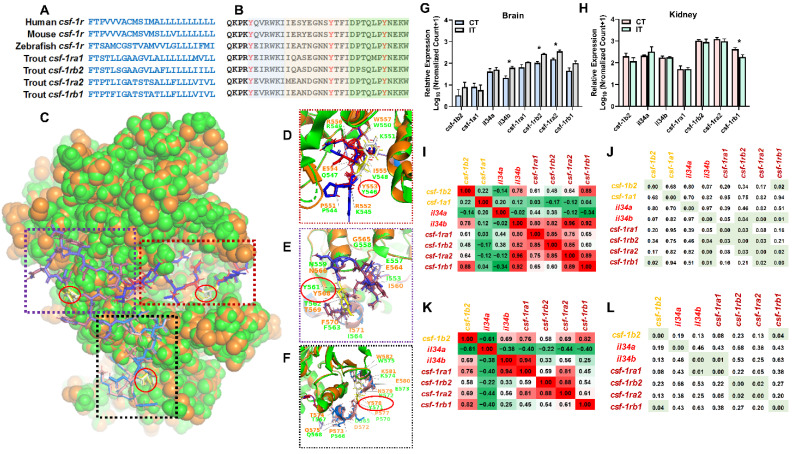
Identification of novel *csf-1r* gene subtypes and characterization of the expression and function. (**A**,**B**) Alignment of novel trout *csf-1r* gene subtypes to teleost and mammalian species. (**A**) Alignment of transmembrane domains of novel trout CSF-1R subtypes to teleost and mammalian species. (**B**) Alignment of juxtamembrane domains of novel trout *csf-1r* gene subtypes to teleost and mammalian species. The JM-B (buried region), JM-S (switch motif), and JM-Z (zipper region) are shaded by different colors and the conserved tyrosine residues are highlighted red. The whole sequence alignment and phylogenetic tree of CSF-1R are shown in Figures S2 and S3. (**C**–**F**) Comparison of the predicted crystal structures of JM-B (**D**), JM-S (**E**), and JM-Z (**F**) between trout and human CSF-1R orthologous. The green illustration shows the human CSF-1R, and the orange illustration shows the trout CSF-1RA1 (LOC100136949). The conserved tyrosine residues are highlighted by a red circle. The comparison model was built via the SWISS-MODEL server (https://swissmodel.expasy.org/, last accessed on 21 December 2021) with human macrophage CSF-1R (3KRJ). (**G**–**L**) The expression profiles of *csf-1* and *csf-1r* systems in brain (**G**) and kidney (**H**). The Pearson’s correlation analysis ((**I**), brain; (**K**), kidney) and *p* value ((**J**), brain; (**L**), kidney) of *csf-1* and *csf-1r* systems in trout in CT and IT. The “*” indicates a significant difference between CT and IT (*p* < 0.05).

Colony stimulating factor 1 receptor (CSF-1R) serves as the receptor of CSF-1 and interleukin-34 (IL-34). The CSF-1R signaling plays an important role in regulating cellular proliferation, differentiation, and survival [48,49,50,51,52]. Based on brain GO enrichment analyses, we identified three *csf-1r* paralogs were significantly changed after *A. salmonicida* infection and enriched in GO terms associated with innate immune and inflammatory responses (GO:0045087 and GO:0006954, Figure 3A). We further searched the genomic data of rainbow trout (GCA_013265735.3), identifying four *csf-1r* paralogs with conserved transmembrane and juxtamembrane domains (Figure 5A,B). Based on the crystal structure of human macrophage CSF-1R (3KRJ), we built a model between trout and human CSF-1Rs via the SWISS-MODEL server (https://swissmodel.expasy.org/, last accessed on 21 December 2021). Trout and human CSF-1R showed relatively conserved amino acid sequences in juxtamembrane domains (Figure 5C–F), including JM-B (buried region), JM-S (switch motif) and JM-Z (zipper region). We also identified the most conserved Tyrosine (Y) in JM-B, JM-S, and JM-Z (Figure 5C–F).

Based on RNA-Seq data, we observed that brain *il-34b*, *csf-1ra2*, and *csf-1rb2* showed up-regulation in infected trout while the kidney *csf-1rb1* showed up-regulation in infected trout (Figure 5G,H). In the brain, the *csf-1b2* exhibited a significantly positive correlation with the *csf-1rb1*, and *il-34b* exerted positive correlation with receptors of *csf-1ra2*, *csf-1rb1*, and *csf-1rb2* (Figure 5I,J). In the kidney, the *csf-1b2* showed a significantly positive correlation with *csf-1rb1*, and *il-34b* showed a significantly positive correlation with *csf-1ra1* (Figure 5K,L).

## 3. Discussion

### 3.1. Key Immune-Related Genes Commonly Identified in the Brain and Kidney

We identified DEGs of *acod1*, *h2-q9*, *ha2q*, and *tapasin* were consistently up-regulated in the brain and kidney. The gene ontology and pathway analyses further revealed these genes play important roles in immunomodulation (Figure 2). The *Acod1* (Aconitate decarboxylase 1, also known as immune-responsive gene 1, IRG1) gene was first identified in mouse macrophage cell line in response to lipopolysaccharide (LPS) stimulation [53]. The recent medical studies indicated that ACOD1 is markedly up-regulated by pathogen infection and serves as a key regulator of inflammation and infection [54,55,56]. In this study, we observed that infected trout showed an ~15 and 2.4-fold increase of *acod1* expressions in the brain and kidney, respectively (Figure 2), which is consistent with previous studies showing that European common carp (*Cyprinus carpio carpio* L.) exerts up-regulated *acod1* expression in responses to LPS [57]. Our enrichment analyses showed up-regulated *acod1* was highly enriched in GO terms, such as immune response (GO:0006955), inflammatory response (GO:0006954), and cellular response to LPS (GO:0071222) (Figure 2), which suggested a conserved immunometabolism reprogram between mammals and teleost. Moreover, we showed that the kidney exhibited higher basal *acod1* expression (~160-fold higher than brain), while the brain showed higher *A. salmonicida*-induced *acod1* expression (~118 versus 34-fold increase), probably suggesting that CNS and peripheral tissues exerted distinct metabolic signatures during basal and activated immune states.

MHC are polymorphic membrane glycoproteins that play an important role in regulating immune functions in response to self and invading antigens [58,59]. The MHC class I molecules present intracellular peptide fragments of the host cells, while MHC class II molecules present the exogenous antigens [60,61,62]. In infected trout, the up-regulated *h2-q9* (LOC110496224) and *ha2q* (LOC110537685) were associated with biological functions of class I and class II histocompatibility and the up-regulated *tapasin* serves as a chaperone for MHC class I assembly [63]. Moreover, these up-regulated DEGs were enriched in KEGG pathways of antigen processing and presentation (ko04612) and/or phagosome (ko04145, Figure 2). Consistent with our results, previous studies showed that Salmonids exhibits crosstalk between MHC class I and II pathways in response to *A. salmonicida* or virus infection [64,65]. Therefore, we might propose that MHC class I could also be activated by cross presentation during pathogen invasion in teleost, which agrees with studies in medical science showing MHC class I pathway can be activated by exogenous antigens via cross presentation [66]. Our data might provide insights for future studies on identification of molecular markers for *A. salmonicida*-resistant trout families due to the fact that MHC polymorphism is associated with resistance/susceptibility to *A. salmonicida* in Salmonids [65,67].

### 3.2. The Pathway Analysis of Neural and Peripheral Immune Modulation

Inflammation represents a cascade of immune responses that aims to protect the body and defend invaders [68]. Although the brain has long been considered as an immune-privileged organ, accumulating studies in recent decades reveal that activation of immune cells in the brain (such as microglial cells and astrocytes) results in neuroinflammation [69,70]. Compared to human and mice studies, studies on fish neuroinflammation (or neuroimmunology) are limited. Our studies showed that the DEGs were significantly enriched in functional pathways associated with pathogen defenses, immune responses, inflammatory regulations, and dysregulated neural functions (Figure 3). Biomedical studies confirmed that neuroinflammation, which could originate from infectious pathogens, contributes to the pathogenesis of neurodegeneration diseases [71,72,73,74]. Studies of psychoneuroimmunology further revealed that alterations in the brain immune state are associated with sickness (or depression) behaviors, including psychomotor slowing, anorexia, and fatigue [75]. For example, meta-analyses of psychoneuroimmunology showed tumor necrosis factor (TNF)-α, interleukin (IL)-1, and IL-6 levels were significantly increased in patients with depression symptoms [75,76,77]. The depression subjects exert overlapped features with symptoms of sickness behavior [75]. Consistently, trout in the IT group showed significantly increased brain *il-6* and *il-6rα* (*il-6* receptor subunit α) expressions and up-regulated enrichments of cellular response to lipopolysaccharide (GO:0071222), cellular response to interleukin-1 (GO:0071347), TNF signaling pathway (ko04668) and Cytokine-cytokine receptor interaction (ko04060, Table S4). Based on these evidences, we proposed that pathogen infection could disturb the homeostasis of immunomodulation and neuromodulation, thus resulting in typical disease characteristics of infected trout (e.g., symptoms of anorexia and lethargy).

Toll-like receptors (TLRs) play important roles in regulating innate immune and the inflammatory responses [78,79]. Multiple members are identified in the TLRs family in mammals, including cell surface receptors (TLR1, TLR2, TLR4, TLR5, TLR6, and TLR10) and intracellularly receptors (TLR3, TLR7, TLR8, TLR9, TLR11, TLR12, and TLR13). TLRs, which serve as pattern recognition receptors, recognize the foreign microbes/molecules, and subsequently trigger the proinflammatory regulation by activating MyD88, NF-κB, and other signaling cascades [80]. Our study showed the transcriptional levels of *tlr2*, *tlr7*, *tlr8*, and *tlr13* were up-regulated in the brain of infected trout (Figure 4 and Figure S1). Moreover, the expression profiles of genes associated with MyD88 and NF-κB signaling pathways were significantly enriched and increased (Figure 3). Our results were consistent with previous studies, showing that TLRs systems in peripheral immune tissues are involved in immunomodulation in Salmonids in response to *A. salmonicida* infection [81,82]. Taken together, these results suggested that the immune responses could trigger inflammation in both brain and peripheral tissues, rather than immune privilege.

Based on previous studies in the biomedical field [74,75,78,79,80] and our RNA-Seq data, we might propose a potential pathway showing that bacterial infection resulted in neuroinflammation (or neuroimmunology) in trout infected by *A. salmonicida*. After infection, trout exert defenses to foreign pathogens and initiate immune responses. TLR2 could detect the extracellular pathogen molecules while the intracellularly pathogen molecules were recognized by TLR7, TLR8, and TLR13. The activated TLRs trigger the downstream MyD88 and NF-κB signaling, enhancing transcriptional levels of genes involved in inflammatory regulation. Neuroinflammation further results in down-regulation of neural development and function (Figure 3).

Consistently with enrichment results of the brain, the up-regulated genes were enriched in immune functions of antigen presentation (ko04145, ko04612), toll-like receptors and TNF signaling (ko04620, ko04668), and complement and coagulation cascades (ko04610, Figure 4, Table S4). The kidney also exhibited significantly up-regulated DEGs associated with endocrine functions (Figure 4, Table S4). The products (cytokines, hormones, and neurotransmitters) and cognate receptors of immune-neuro-endocrine axis coexist in cells, thus playing an important role in regulating and recovering homeostasis in response to pathogen and stress in both mammals and teleost [83,84,85,86,87]. The kidney showed up-regulated signaling pathways associated with estrogen, prolactin, and insulin signaling (ko04915, ko04917, ko04910), insulin secretion (ko04911), and thyroid hormone synthesis (ko04918, Figure 4, Table S4), which agreed with previous studies showing endocrine products, such as prolactin and estrogen, and exerts immune-regulatory effects on fish (reviewed in [84,88,89]).

### 3.3. Identification of Novel Genes Associated with Immunomodulation

CSF-1 is an important regulator in regulating macrophage differentiation and survival, which plays a vital role in immunomodulation [90,91]. The *csf-1* gene is extensively expressed in multiple species including mammals, birds, and teleost [92,93,94]. Only one *csf-1* gene is identified in most mammals, while teleost (excepting Salmonids) exerts two *csf-1* (*csf-1a* and *csf-1b*) copies due to the additional whole genome duplication (three rounds of genome duplication, also termed as teleost-specific WGD) [95,96]. In this study, we identified four *csf-1* paralogs in trout, which is consistent with the fact that Salmonids’ ancestry exerts four rounds of genome duplication [97]. The CSF-1R, a glycoprotein encoded by the *c-fms* proto-oncogene [51], serves as the receptor of CSF. Consistently, we identified four *csf-1r* paralogs in rainbow trout. Phylogenetic analysis indicated orthologous relationships of *csf-1* and *csf-1r* between mammalian and teleost equivalents (Figure S3), potentially suggesting the conserved functions in immunomodulation.

In this study, *A. salmonicida* infection resulted in significantly increased *csf-1ra2* and *csf-1rb2* expressions in the brain and decreased *csf-1rb1* expression in the kidney, suggesting the functional diversity of *csf-1r* paralogs and the potential tissue-specific expressions. Further enrichment analyses showed up-regulated brain *csf-1r* genes were involved in up-regulated GO terms associated with innate immune response (GO:0045087) and inflammatory response (GO:0006954, Figure 3 and Table S4). Our results agree well with previous studies showing that the CSF-1 system is involved in immunoregulatory property in teleost [98,99]. Biomedical studies confirmed that both CSF-1 and interleukin-34 (IL-34) activate human CSF-1R. In this study, we observed brain *il-34b* was up-regulated after the *A. salmonicida* infection, which is consistent with the expression pattern of *csf-1ra2* and *csf-1rb2*. Moreover, the *il-34* expression was positively correlated with *csf-1r* expressions (Figure 5). Further studies might focus on pharmacological characteristics of *il-34* and *csf-1r* via in vitro studies.

## 4. Materials and Methods

### 4.1. Ethics Statement

All experiments were conducted in accordance with guidelines of Animal Research and Ethics Committee of Ocean University of China (Permit Number: 2014201), the National Institutes of Health Guidelines for the Care and Use of Laboratory Animals (NIH Publications NO. 8023, revised 1987). Our research did not involve endangered or protected species. In this study, trout juveniles were immature, and the effect of gender was not considered.

### 4.2. Experiment Design and Sample Collection

Rainbow trout (~10 g and ~8 cm) were obtained from trout farm in Linqu, Shandong Province, and then acclimated for 7 days in individual aquariums at ~17 °C with the photoperiod of 12:12 (hours) of light and dark. During bacterial challenge, trout were randomly classified into two groups as control trout group (CT) and infected trout group (IT), respectively. IT contained two replications and each replication had 20 individuals (n = 20). Based on our previous study [100], trout of IT were intraperitoneally injected with 0.2 mL of *A. salmonicida* (1 × 10^8^ CFU/mL), while trout in CT were injected by an equal volume (0.2 mL) of phosphate buffer solution (PBS). Furunculosis symptoms (Figure S4) were observed at 48 h after infection; therefore, trout in both the CT and IT groups were euthanized by MS-222 (35–45 mg/L) and then sampled at 48 h. The brains and kidneys were collected after washing with PBS (remove blood and fat cells) and then stored at −80 °C for further analyses. Two individuals in IT were pooled as one sample to reduce the individual variations for further analyses.

### 4.3. RNA-Seq Analysis

Total RNA of the brain and kidney was extracted using TRIzol reagent (Invitrogen, Carlsbad, CA, USA), and the RNA quality was evaluated by NanoDrop ND-1000 (Thermo Fisher Scientific, Wilmington, DE, USA) and 1% agarose gel electrophoresis. One transcriptome library was constructed by pooling equal quantities of RNA from two individuals. A total of 12 libraries (2 tissues x 3 replicated samples × 2 treatment groups) were constructed via TruSeq^TM^ RNA Sample Prep Kit (Illumina, San Diego, CA, USA). The Illumina Hiseq X Ten platform (OE biotech Co., Ltd., Shanghai, China) was used to generate 150 bp paired-end raw reads. Raw data were processed using the Trimmomatic [101]. The clean reads were obtained by removing reads with low quality or reads containing poly-N. The clean reads were then mapped to the reference genome of rainbow trout (GCA_013265735.3) using histat2 [102]. The sequence reads are available at the NCBI sequence read archive (SRA), accession number PRJNA753277.

The Fragments Per kb Per Million Reads (FPKM) value of each gene was calculated using cufflinks, and the read counts of each gene were obtained by htseq-count. Differentially expressed genes (DEGs) were identified by the DESeq2 R package [103,104] and the DEGs were identified as *p*-value < 0.05 and |log_2_(fold change)| > 1. Gene Ontology (GO) and Kyoto Encyclopedia of Genes and Genomes (KEGG) pathway enrichment analyses of DEGs were evaluated, primarily focusing on functional/signaling pathways associated with immunomodulation and neural functions.

### 4.4. Identification of Novel Immune Genes Based on RNA-Seq Data

The RNA-Seq data showed that macrophage colony stimulating factor 1 (*csf-1*, also known as *cfms*) paralogs and their cognate receptors (*csf-1r*) were significantly involved in immune responses of trout in response to *A. salmonicida* challenges. To identify the novel paralogs of *csf-1* and *csf-1r*, the whole genomic sequence database (GCA_013265735.3) of rainbow trout was researched using the TBLASTN program. The CSF-1 and CSF-1R full-length amino acid sequences of human and zebrafish were used as the queries with an E-value of 1 × 10^−5^. The TBLASTN and Clustal W were used to remove redundant sequence(s), thus generating initial candidate sequences for further analysis.

Phylogenetic analysis was conducted to validate the annotation of the potential CSF-1 and CSF-1R paralogs in trout. The amino acid sequences of human (*Homo sapiens*), mice (*Mus musculus*), zebrafish (*Danio rerio*), medaka (*Oryzias latipes*), channel catfish (*Ictalurus punctatus*), Atlantic salmon (*Salmo salar*) and other teleost were used for the construction of the phylogenetic tree. An alignment of multiple amino acid sequences was performed using ClustalW. The neighbor-joining (NJ) method and Jones–Taylor–Thornton (JTT) model were used for conducting phylogenetic and molecular evolution analysis by using MEGA 7.0 software. Bootstrap tests with 1000 replications were used to test the phylogenetic tree and gaps were removed via pairwise deletion. Comparison between trout and mammalian CSF-1R (human, 3KRJ) were generated with the SWISS-MODEL (https://swissmodel.expasy.org/, last accessed on 21 December 2021) and PyMOL software package.

### 4.5. Validation of RNA-seq Data by qPCR

**Figure 6:**
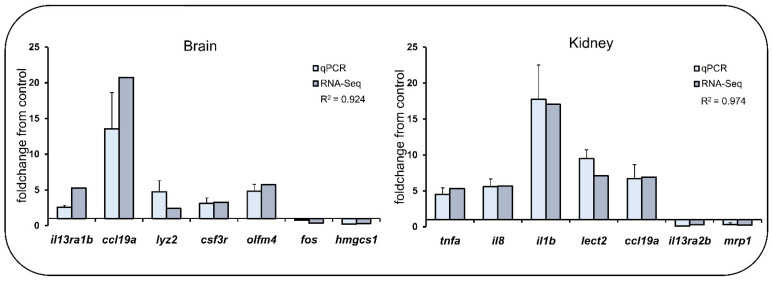
RNA-Seq validation. Comparison of gene expression levels between RNA-seq and qPCR. The relative expression values were normalized to *β*-actin gene expression for qPCR analysis. Foldchange refers to the fold-change between the CT and IT.

Thirteen DEGs were selected for qPCR analysis. They were shown in Figure 6 and Table S1. The specific amplification of each primer pairs was validated, and the amplification efficiency was calculated as E (%) = (10^(−1/slopes)^ − 1) × 100. The *β-actin* was used as an internal reference gene [100]. The qPCR was performed on StepOnePlus^TM^ Real-time PCR system (Applied Biosystems, Carlsbad, CA, USA). The reaction volume was 10 μL, containing 1 μL cDNA, 5 μL SYBR^®^ FAST qPCR Master Mix, 0.2 μL forward (reverse) primer, and 3.6 μL RNAase-free water. The qPCR was performed by following the following program: 95 °C for 30 s, 40 cycles of 95 °C for 10 s and Tm for 30 s, followed by 72 °C for 30 s. The relative expression levels of mRNA were calculated using the comparative 2^−ΔΔCT^ method [105].

### 4.6. Statistical Analysis

Based on published medical and fishery studies [106,107] the RNA-Seq data (count normalized by DESeq2 [104]) were uploaded to the websites of MetaboAnalyst and NetworkAnalyst (https://www.xialab.ca/tools.xhtml, last accessed on 21 December 2021) for data processing and analyses [108]. The data were analyzed by principal components analysis (PCA), loading plots and heatmaps via a multivariate analysis module of MetaboAnalyst. The univariate analyses of gene expression were analyzed by GraphPad Prism 8.0. One-way analysis of variance (ANOVA) was used to evaluate the effect of *A. salonmicida* challenge on gene expressions of trout in CT and IT. Means of gene expressions were further compared by Tukey’s multiple range tests when significant differences were observed by one-way ANOVA. The differences were considered statistically significant when the *p* < 0.05. Results were expressed as mean ± standard error (means ± S.E.). The correlation analysis of gene expression was investigated by MetaboAnalyst and GraphPad Prism 8.0 via Pearson’s correlation analysis.

## 5. Conclusions

**Figure 7:**
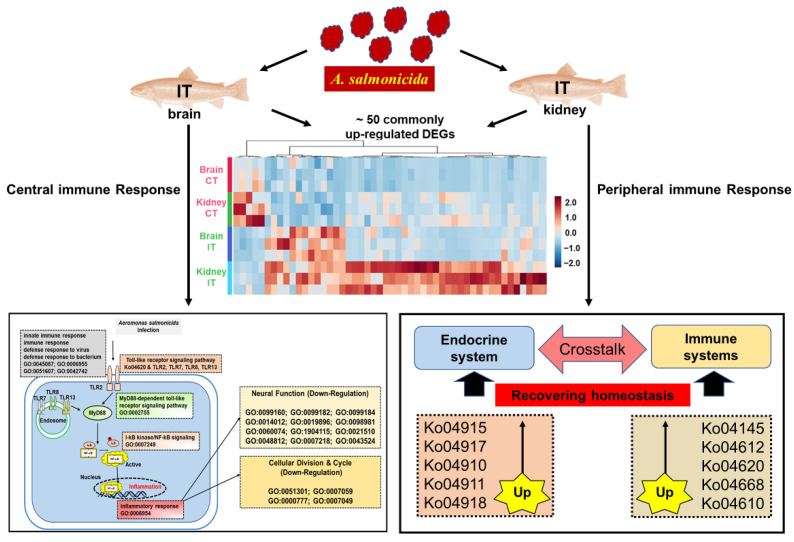
Putative pathways involved in central and peripheral responses based on RNA-Seq signatures.

The pairwise comparison between trout of the CT and IT groups (Figure 7) showed that the kidney and brain shared ~50 up-regulated DEGs with signaling pathways including antigen presentation (ko04145, ko04612), toll-like receptors and cytokine functions (ko04620, ko04668), and complement and coagulation cascades (ko04610). This evidence confirmed that *A. salmonicida* infections resulted in homogeneously immune effects on both central and peripheral tissues, rather than CNS immune privilege. In the brain, the up-regulated DEGs were associated with activation of the toll-like receptor signaling pathways, thus triggering neuroinflammation and dysregulated neuro functions. Therefore, the typical disease characteristics of infected trout, such as symptoms of anorexia and lethargy, could be partly attributed to the dyshomeostasis between immunomodulation and neuromodulation in brain. In addition, kidneys also showed up-regulated endocrine networks involved in estrogen, prolactin, and insulin signaling pathways and thyroid hormone synthesis, supporting the bi-directional crosstalk between endocrine and immune systems in response to pathogen infection.

## Data Availability

Data are contained within the article.

## Supplementary Materials

The following are available online at https://www.mdpi.com/article/10.3390/ijms23031340/s1.
