# Supplementary material for: Transcriptional Signatures of Immune, Neural, and Endocrine Functions in the Brain and Kidney of Rainbow Trout (Oncorhynchus mykiss) in Response to Aeromonas salmonicida Infection
Source: Int J Mol Sci. 2022 Jan 25;23(3):1340. doi: 10.3390/ijms23031340 (PMC8835788; doi:10.3390/ijms23031340)
Supplement: Supplementary file 1 [file ijms-23-01340-s001.zip › ijms-1543762-supplementary.pdf]

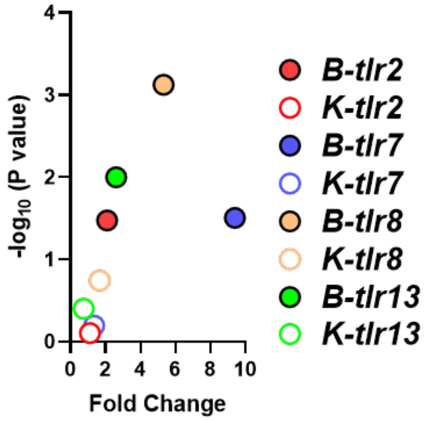

Figure S1. Expression profiles of tlr2, tlr7, tlr8 and tlr13. B indicates brain and K indicates kidney.

```
                                                         signal peptides
Human            --------------------------------------------MGPGVLLLLLVATAWHGQGIPVIEPS-----VPELV     31
Mouse            --------------------------------------------MELGPPLVLLLATVWHGQGAPVIEPS-----GPELV     31
Zebrafish        ------------------------------------------MFFALLFLIGILLGQVQG-WSEPRIRLSSGALAGTDVI     37
Trout csf-1ra1   ------------------------------------------MELYLAFLLGILPTAAQ-EWRRPVIKLNSEVVVGSEVV     37
Trout csf-1ra2   MLDPYSLVRSDLTTVLKTSTDEITTLRDIQTYSQTQSWKRSRMVLYLAFLLGILPTAAQAQWRHPVIKLNSQLVVGSEVV     80
Trout csf-1rb2   -------------------------------------------MLFLALFLGIMLC-TAQERSPPVIRLNSVLLREAERT     36
Trout csf-1rb1   -------------------------------------------MLILALLLGIMVCCTAQEQSPPVIKLNSVVLREAVWT     37

Human            VKPGAT-VTLRCVGNGSVEWDGPPSPHWTLYSDGSSSILSTN--NATFQNTGTYRCTEP---GDPLGGSAAIHLYVKDPA     105
Mouse            VEPGET-VTLRCVSNGSVEWDGPISPYWTLDPESPGSTLTTR--NATFKNTGTYRCTEL---EDPMAGSTTIHLYVKDPA     105
Zebrafish        LESGSP-LQLVCEGDGPVTFLPRLAKHKRYISKEVGKIRSFRVEKTTVDFTGTYKCVY--MNGNDSNLSSSVHVFVRDSR     114
Trout csf-1ra1   LNPGTP-LVLRCEGDGPVNWLTRLSKHKSLISKGNGRVRTFKVDRPSAEHTGTYKCEYTSVNVKVRDLFSTVHVYVKDPD     116
Trout csf-1ra2   LNPGTP-LVLRCEGDGPVNWLTRLAKHKRFISKVKGRVRTFRVERPSAEHTGTYKCEYTSVNVKGQNLFSTVHVYVKDPD     159
Trout csf-1rb2   LLLGTS-FTLTCEGDSTATWSTTAFK-RR--NSKQGSVITTR--YLTADYTGTYMCVYD----NQPDLFSEVHIYVKDPN     106
Trout csf-1rb1   IPVGTSSFTLTCEGYSTVTWSTTAFKLMR--KTRLGSVITTR--RLTVDYTGTYKCMYE----NQPDLLSEVHIYVKDPH     109

Human            RPWNVLAQEVVVF--EDQDALLPCLLTDPVLEAGVSLVRVRGRPLMRHTNYSFSPWHGFTIHRAKFIQSQDYQCSALMGG     183
Mouse            HSWNLLAQEVTVV--EGQEAVLPCLITDPALKDSVSLMREGGRQVLRKTVYFFSPWRGFIIRKAKVLDSNTYVCKTMVNG     183
Zebrafish        VLFVSPSTSLRYVRKEGEDLLLPCLLTDPEATD-FTFRMDNGSAAPYGMNITYDPRKGVLIRNVHPGFNADYICCARIGG     193
Trout csf-1ra1   SLFWTSSASLRVVRKEGEDHLLPCLLTDPEATD-LGLRMDNCTFVPPGMNYTADPRRGILIRNLHPSYNADYVCSAKLHG     195
Trout csf-1ra2   SLFWTSSTSLSVVRKEGENYLLPCLLTDPEATD-LGLRMDNCTSVPPGMNYTADPRRGILIRNLQPSYNADYVCSAKLHG     238
Trout csf-1rb2   NVLVTPRNMRDV--EEGGDLLL-CQLTDPSATD-MSLHMANGDPTPPDMNYTVNPRRGILIRHLLTSHSANYICSAKING     182
Trout csf-1rb1   NILVTPRTLGDV--KEGSDVLL-CQLTNPIATD-LSVRMANGDPTPPDMNYTVNPKRGILIRYLLTSHSADYVCSAKING     185

Human            RKVMSISIRLKVQKVIPGPPALTLVPAELVRIRGEAAQIVCSASSVDVNFDVFLQHNNTK---LAIPQQSDFHNNRYQKV     260
Mouse            RESTSTGIWLKVNRVHPEPPQIKLEPSKLVRIRGEAAQIVCSATNAEVGFNVILKRGDTK---LEIPLNSDFQDNYYKKV     260
Zebrafish        AEKVSKIFSINIIQRLRFPPYVYLKRNEYVKLVGERLQISCTTNNPNFYYNVTWTHSSRMLPKAEEKSTMEG-D-RLAIE     271
Trout csf-1ra1   VERTSKTFNLNIIQRLRFPPYVFLEKDEYVHIVGEKLSIHCTTHNPNFNYNVTWNYSSKKRFTIEQKVQSVD-SNRLDIE     274
Trout csf-1ra2   VERTSKAFSINIIQRLRFPPYVFLEKDEYVRIVGEKLRIHCTTHNPNFNYNVTWNYSSKKRSTIEENVQSKD-S-RLDIE     316
Trout csf-1rb2   VTTRSKDITINVVQRLRWPPSVLIEVDGYVSIVGEELRIPCITSNPNHFYNVTWKHSSLKALNFFQTVIQ---DKQVHIT     259
Trout csf-1rb1   VTKVSKVVPIFVIQSLRLPPSVLIEVNGYVRIVGEQLLIPCITSNPNHFYNVTWKHSSLKVLDFSHKMIQGDQSNQVHIT     265

Human            LTLNLDQVDFQHAGNYSCVASNVQGKHSTSMFFRVVESAYLNLSSE-------QNLIQEVTVGEGLNLKVMVEAYPGLQG     333
Mouse            RALSLNAVDFQDAGIYSCVASNDVGTRTATMNFQVVESAYLNLTSE-------QSLLQEVSVGDSLILTVHADAYPSIQH     333
Zebrafish        SILTIPSVQLSHTGNITCTGQNEAGANSSTTQLLVVEEPYIRLSPKLSSKLTHRGLSIEVSEGDDVDLGVLIEAYPPLTS     351
Trout csf-1ra1   SILTIPVVDQSDTGNITCIGTNEAGVNKSTTSLIVVEEAYIRLSPQLSSKLAHQGLSIDVNEGEDLKLSVLIEAYPQIIG     354
Trout csf-1ra2   SILTIPAVDQSDTGNITCTGTNEAGVNSSATYLLVVEEPYIRLSPQLSSKLAHQGLSIEVNEGEDLKLCVLIEAYPQIIA     396
Trout csf-1rb2   STVTIPAVTMSDTGNFTCTAMNEAGASRSTIYLEVVDEPYIRFIPRLSPNLYQNGSLVNVKEGENLEISILIEAYPQIKK     339
Trout csf-1rb1   SSVTIPAVAMSHTGNFTCTAMNEAGFNSSTTYLQVVDKPYMRLIPRLSPDLYLNGSLVEVNEGENLEINIQIEAYPQIME     345

Human            FNWTYLGPFSDHQPEPKLANA-TTKDTYRHTFTLSLPRLKPSEAGRYSFLARNPGGWRALTFELTLRYPPEVSVIWTFIN     412
Mouse            YNWTYLGPFFEDQR--KLEFI-TQRAIYRYTFKLFLNRVKASEAGQYFLMAQNKAGWNNLTFELTLRYPPEVSVTWMPVN     410
Zebrafish        HKWET--PTSHNASLPENRFF---NHNDRYEALLLLKRLNFEEIGQYTLNVKNSMKSASITFDIKMYTKPVARVKWENVT     426
Trout csf-1ra1   QHWAT--PTA--SSTQEQTFT---RYINRYSATLLLKRMIAQEQGQYTFYAKSPMANASITFQIQMYQRPVAVVRWENIT     427
Trout csf-1ra2   QSWDT--PTS--SPTQEHIFT---RYNNRYSATLLLKRMIAQEQGQYTFYARSAMANASITFQVQMYQKPVAVVRWENIT     469
Trout csf-1rb2   HWWDV--PMSHSQNISTHGDTWTAQDNNRYESSLLLHRVRSEERGQYTLHTRSTRLNSSITFNIQVYQKPSAMLRLKNST     417
Trout csf-1rb1   QWWDI--PMSHNHNISTHDDTWAVRFNNRYESSLLLQRVRSEERGQYTLHTRSTCLNGSITFNVQVYQKPSAMVLLKNVT     423

Human            GSGTLLCAASGYPQPNVTWLQCSGHTDRCDEAQVLQVWD---DPYPEVLSQEPFHKVTVQSLLTVETLEHNQTYECRAHN     489
Mouse            GSDVLFCDVSGYPQPSVTWMECRGHTDRCDEAQALQVWN---DTHPEVLSQKPFDKVIIQSQLPIGTLKHNMTYFCKTHN     487
Zebrafish        ---TLSCRSYGYPAPSILWYQCTGIRTTCPENTTDLQ---PIQTQTVEFQKESFGAVGVESVLTVGP-NRRMTVVCVAFN     499
Trout csf-1ra1   ---TLTCTSFGYPAPIILWYQCSGIRTTCNENATGLQMPAPLLAQTVEVQREEYGVVGVQSVLTMEPSSHRLTVECVAFN     504
Trout csf-1ra2   ---SLMCTSFGYPAPIILWYQCSGIRATCNENNTGLQMPAPLLAQTVEVQREEYGPVEVESVLTVGPSNRRMTVECVAFN     546
Trout csf-1rb2   ---TLICTSSGYPAPTILWYQCPGIQSACDADNDTVEVQ-PLFTSTMEVQSE----------LTLSPSTEEFTVECVTFN     483
Trout csf-1rb1   ---TLTCTASGYPAPTILWYQCPGIQNTCDGDNDTVEVQ-PLLTSTMEVQSE----------VTLSPSSMEVTVECVTFN     489
```

```
transmembrane domains
Human           SVGSGSWAFIP-ISAGAHTHPPDEFLFTPVVVACMSIMALLLLLLLLLLYKYKQKPKYQVRWKIIESYEGNSYTFIDPTQ   568
Mouse           SVGNSSQYFRA-VSLGQSKQLPDESLFTPVVVACMSVMSLLVLLLLLLLYKYKQKPKYQVRWKIIERYEGNSYTFIDPTQ   566
Zebrafish       LVGQGSDTFSMEV---------SDQIFTSAMCGSTVAMVVLGLLLIFMIYKYKQKPRYEIRWKIIEATNGNNYTFIDPTQ   570
Trout csf-1ra1  LVGVGKDTFAMDV---------SNIMFTSTLLGAAGVLALLLLLLMVLLYKYKQKPRYEIRWKIIQASEGNNYTFIDPTQ   575
Trout csf-1ra2  LAGVGKDTFAMEV---------SDKVFTSTLSGAAGVLAFLILLLIILLYKYKQKPRYEIRWKIIQASDGNNYTFIDPSQ   617
Trout csf-1rb2  FAGKNRDIFISHVVAATSKTFVTSELFTPTLIGATSTSALLFLLLVIVLYKYKQKPKYEVRWKIMEANDGNNYTFIDPTQ   563
Trout csf-1rb1  LVGKERDIFVLRVPAATLPTFATPKLFTPTFIGATSTATLLFLLLVIVLYKYKQKPKYEVRWKIIEANDGNNYTFIDPTQ   569

Human           LPYNEKWEFPRNNLQFGKTLGAGAFGKVVEATAFGLGKEDA-VLKVAVKMLKSTAHADEKEALMSELKIMSHLGQHENIV   647
Mouse           LPYNEKWEFPRNNLQFGKTLGAGAFGKVVEATAFGLGKEDA-VLKVAVKMLKSTAHADEKEALMSELKIMSHLGQHENIV   645
Zebrafish       LPYNEKWEFPRDKLKLGKTLGAGAFGKVVEATAYGLGKEDN-ITRVAVKMLKASAHPDEREALMSELKILSHLGQHKNIV   649
Trout csf-1ra1  MPYNEKWEFPRDKLKLGKILGAGAFGKVVEATAYGLGEDDN-AMRVAVKMLKARAHSDEREALMSELKILSHLGQHKNIV   654
Trout csf-1ra2  LPYNEKWEFPRDKLKLGKILGAGAFGKVVEATAYGLGEDDN-AIRVAVKMLKARAHSDEREALMSELKILSHLGQHKNIV   696
Trout csf-1rb2  LPYNKKWEFPRDKLRLGQILGAGAFGKVVEATAYGLGTDDNMTTRVAVKMLKPSAHSEEREALMSELKILSHLGCHDNIV   643
Trout csf-1rb1  LPYNKKWEFPRDKLRLGQILGAGAFGKVVEATAYGLGTGDNMTTRVAVKMLKPSAHSEEREALMSELKILSHLGSHDNIV   649

Human           NLLGACTHGGPVLVITEYCCYGDLLNFLRRKAEAMLGPSLS-PGQD-----PEGGVDYKNIHLEKKYVRRDSGFSSQGVD   721
Mouse           NLLGACTHGGPVLVITEYCCYGDLLNFLRRKAEAMLGPSLS-PGQD-----SEGDSSYKNIHLEKKYVRRDSGFSSQGVD   719
Zebrafish       NLLGACTHGGPVLVITEYCCHGDLLNFLRSKAENFLNFVMTIPN------FPEPMTDYKNVSTERMFVRSDSGISSTCSD   723
Trout csf-1ra1  NLLGACTQAGPVLVITEYCSHGDLLNFLRHKQETFLNFVMNIPA------VPEETSDYKNLCEGKQFIRSDSGISSVCSD   728
Trout csf-1ra2  NLLGACTQAGPVLVITEYCSHGDLLNFLRHKQETFLNTVMNMPE------VPEETSDYKNVCEEKQFIRSDSGISSVSSD   770
Trout csf-1rb2  NLLGACTQGGPMLMITEYCSHGDLLNFLRGKAKLFLDSILSGPG------IPGNSDHYKNTCSQESRVRSDSGISCSSSD   717
Trout csf-1rb1  NLLGACTQGGPMLMITEYCSYGDLLNFLHGKAKLFLYSIPRGPGTPEVPRVPENHDHYKNTCAQESRVRSDSGISCSSSD   729

Human           TYVEMRP----VSTSSNDSFSEQDLDKEDGRPLELRDLLHFSSQVAQGMAFLASKNCIHRDVAARNVLLTNGHVAKIGDF   797
Mouse           TYVEMRP----VSTSSSDSFFKQDLDKEASRPLELWDLLHFSSQVAQGMAFLASKNCIHRDVAARNVLLTSGHVAKIGDF   795
Zebrafish       HYLDMRPVTSRPTNSALDSSSECQE---DSWPLDMDDLLRFSSQVAQGLDFLAAKNCIHRDVAARNVLLTNSRVAKICDF   800
Trout csf-1ra1  SYLEMRP-GPQPVNSSLDSV--CEDGGPDSWPLDMEDLLRFSYQVAQGLDFLAAKNCIHRDVAARNVLLTDLHVAKICDF   805
Trout csf-1ra2  SYLEMRP-GPQPVNSSLDSL--CEEGGPDSWPLDMDDLLRFSYQVAQGLDFLAAKNCIHRDVAARNVLLTDRRVAKICDF   847
Trout csf-1rb2  NYQDMHP-ARRPKYCPMGSL--CEDPETDTWLLDMEDLLRVSYQVAQGMDFLASKNCIHRDAAARNVLLTDGHVAKICDF   794
Trout csf-1rb1  NFLDTHP-AQRPKHCPMGSL--CEDPETGTWSLDIEDLLRFSSQVAQGMDFLASKNCIHRDVAARNVLLTDGRVAKICDF   806

Human           GLARDIMNDSNYIVKGNARLPVKWMAPESIFDCVYTVQSDVWSYGILLWEIFSLGLNPYPGILVNSKFYKLVKDGYQMAQ   877
Mouse           GLARDIMNDSNYVVKGNARLPVKWMAPESIFDCVYTVQSDVWSYGILLWEIFSLGLNPYPGILVNNKFYKLVKDGYQMAQ   875
Zebrafish       GLARDIMNDSNYVVKGNARLPVKWMAPESIFECVYTVQSDVWSYGIMLWEIFSLGKSPYPNILVDSKFYKMIKCGYQMSR   880
Trout csf-1ra1  GLARDIMNDSNYVVKGNARLPVKWMAPESIFDCLYTVQSDVWSYGILLWEIFSLGKSPYPSILVDTKFYNMIKCGYQMSR   885
Trout csf-1ra2  GLARDIMNDSNYVVKGNARLPVKWMAPESIFDCLYTVQSDVWSYGILLWEIFSLGKSPYPSILVDTKFYKMIESGYQMSR   927
Trout csf-1rb2  GLARDIENDSNYVVKGNARLPVKWMSPESIFDCVYTVQSDVWSYGILLWEVFSLGKSPYPNIVVDTRFYKMIKDGCHMSQ   874
Trout csf-1rb1  GLARDIENDSNYVVKGNARLPVKWMAPESIFECVYTVKSDVWSYGILLWEIFSLGKSPYPNVVVDTRFYKMIKDGCYMSQ   886

Human           PAFAPKNIYSIMQACWALEPTHRPTFQQICSFLQEQAQE--DRRE-------RDYTNLPSS-------SRSGGSGSSSSE   941
Mouse           PVFAPKNIYSIMQSCWDLEPTRRPTFQQICFLLQEQARL--ERRD-------QDYANLPSSGGSSGSDSGGGSSGGSSSE   946
Zebrafish       PDFAPPEMYTIMKMCWNLDAAERPTFSKISQMIQRMLGETSEQQD------TQEYKNIP-TEAEAEQQLESC--------   949
Trout csf-1ra1  PDFAPPEMYTIMKMCWNLEPTERPTFSKISQLIERLLGEEPERPDQCSTLPSQQHQNIQLQQDMMVEELELCDDNDE---   964
Trout csf-1ra2  PDFAPPEMYTIMKMCWNLEPTERPTFSKISQLIERVLGEQPEHLD-------QQYQNIQ-QQDMVIEELEPCDDDDDKTK   999
Trout csf-1rb2  PDFAPPEIYTIMKMCWNMEPTVRPTFSTIGQLIQSLL---PDQPD-------QTYRNVQ---DKTPRQESGEQSGQAKI-   940
Trout csf-1rb1  PDFAPPEIYTIMKMCWNLEPTERPAFSTIGQLIQRLL---PDQPD-------HTYRNVQ---DKTPQQGAGEQGDPDKT-   952

Human           LEEESSSEHLTCCEQGDIAQPL-LQPNNYQFC                                                   972
Mouse           PEEESSSEHLACCEPGDIAQPL-LQPNNYQFC                                                   977
Zebrafish       HEDESFET---SCDQEEEDQPL-MKPNNYQFC                                                   977
Trout csf-1ra1  CCDGSCDQ---SCEHEEEEQPL-VKTNNYQFC                                                   992
Trout csf-1ra2  FCDGSCDQ---SCEHEEEEQPL-MKTNNYQFC                                                   1027
Trout csf-1rb2  --SEDCDQ---TLNHEEEEQPLMMRNNNYQIC                                                   967
Trout csf-1rb1  --NGDCEQ---TLNQEGEEEPL-MKNNNYQFC                                                   978
```

Figure S2. The whole sequence alignment of CSF-1R among mammals and teleosts. The signal peptides, transmembrane domains and conserved tyrosine residues are highlighted.

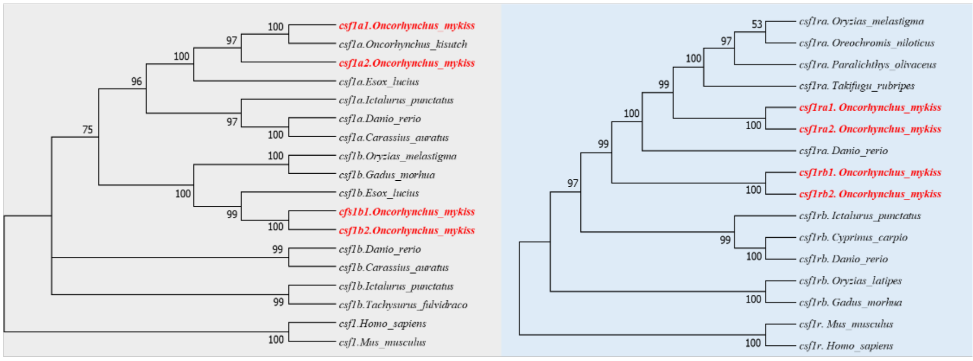

Figure S3. Evolutionary relationships of CSF-1 and CSF-1R.

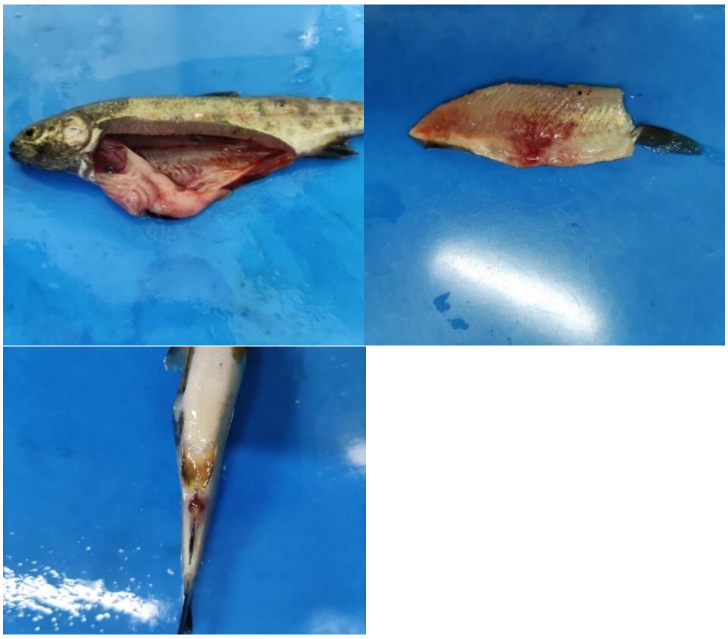

Figure S4. Furunculosis symptoms: Intestine hyperaemia; Muscle fester; Anus become red and turgescence, extrudes out.

Table S1. The primers for qPCR validation.

Primer name	Forward primer (5′-3′)	Reverse primer (5′-3′)
tnfα	GGGGAACCAAATCCTCAT	GGACTCAGCATCACCGTAG
il1β	AGGACCTGCTCAACTTCTT	ACCCAGCACTTGTTCTCA
il8	CTCAAGATACAAACCAAATAAG	AACCGACTGTCTTTTCAC
olfm5	AAAACCTGCCTTCTACAGCAAA	TACCCACGGCACACTGAAAC
ccl19a	CTCTGACAGGGACCTTAGGGA	TCACTACCAGGAGGGGAGTC
csf3r	GGAACGCTGCAGGGACAT	CACACCACCACATAGCCTGT
lyz2	GCATCCGGAATGGATGGCTA	CAGCGGATACCACAGACGTT
mrp1	TGGAGGGTTGGTTATTAGG	AGGAAGAAAGGTCCGAAG
lect2	GCTGTTCTTTTGTTTACTGTGGTG	TGTCCTTGTCCCCATCTGTC
hmgcs1	CCTGGTCAACTCCACCACAG	TCCCCATTGCTAACGGTGAC
il13ra2b	AGCAGATGTTGAAGCAGAGGAATG	ACACAAAACCAGGAGGTTGC
il13ra1b	ACCCTGGTGAATCCCACAAA	ACACACCCGGAAGGTTTTCT
fos	GCCTGAGGAGGAGGAGAAGA	CCATGGGAGGGAGAGATGGA
β actin	GATGGGCCAGAAAGACAGCTA	TCGTCCCAGTTGGTGACGAT

Table S2. The DEG lists of Figure 2 (Up-regulated in IT).

Gene id (NCBI)	Base Mean CT	Base Mean IT	log_2FC	*p* val	padj	Gene symbol
			**Brain**			
LOC100136187	0.000	49.699	Inf	0.00000	0.00000	
LOC118941484	0.000	14.976	Inf	0.00012	0.05927	
LOC118964931	0.000	126.631	Inf	0.01658	0.83024	*saa3*
LOC118936275	0.305	46.651	7.259	0.00000	0.00000	
acod1	0.305	35.963	6.883	0.00090	0.21826	*acod1*
LOC110530481	0.374	37.718	6.658	0.00117	0.23514	
LOC110496224	34.882	1151.884	5.045	0.02758	0.94987	*(h2-q9)*
LOC110509875	1.431	39.826	4.799	0.00000	0.00068	

LOC110536048	0.678	16.068	4.566	0.00079	0.20339	*sds*
LOC110518069	0.647	14.632	4.500	0.00203	0.33134	*gvin1*
ccl19a.1	0.715	14.847	4.375	0.04152	1.00000	*ccl19*
LOC110531529	1.399	23.232	4.053	0.00175	0.29805	*mefv*
LOC110497998	86.342	1155.330	3.742	0.00000	0.00000	
LOC110510403	2.239	29.482	3.719	0.00437	0.48474	*mfap4*
si:ch211-284e13.9	1.020	11.138	3.449	0.01890	0.86259	
tymp	2.109	21.549	3.353	0.00082	0.20807	*tymp*
LOC118936255	26.389	263.495	3.320	0.03877	1.00000	
LOC110488725	1.057	9.914	3.229	0.03087	0.97010	*ffar3*
LOC110538203	4.397	36.363	3.048	0.00299	0.41037	*c1ql2*
lect2	9.238	74.298	3.008	0.00000	0.00013	*lect2*
LOC110531659	1.399	10.404	2.894	0.03694	0.99944	*gvin1*
LOC118964557	36.875	232.430	2.656	0.00001	0.01288	
LOC110519256	2.115	12.174	2.525	0.03812	1.00000	*calhm6*
LOC110537685	2.414	12.678	2.393	0.04499	1.00000	*h2-aa (ha2q)*
LOC110528546	46.779	207.095	2.146	0.01160	0.72429	*rnf213a*
LOC110498757	11.875	49.649	2.064	0.00091	0.21826	*fmnl1*
LOC110536323	4.236	17.514	2.048	0.03997	1.00000	*ggh*
LOC110525408	18.276	69.979	1.937	0.00056	0.16298	*gda*
LOC110510228	31.045	109.693	1.821	0.00001	0.01077	
LOC110507168	10.534	36.403	1.789	0.00528	0.51532	
hsp47	100.960	332.702	1.720	0.02298	0.90994	*serpinh1*
LOC110528445	29.766	97.128	1.706	0.00578	0.53427	*ifi44*
LOC110495727	22.788	70.774	1.635	0.02985	0.96324	*ifi44l*
LOC110518874	41.177	125.093	1.603	0.00009	0.04523	*ube2d4*
LOC110533568	7.980	23.959	1.586	0.04443	1.00000	*derl2*
LOC110536252	50.895	145.633	1.517	0.00230	0.35705	*s100a1*
LOC110533271	27.138	75.887	1.484	0.00173	0.29805	
LOC110524047	57.086	153.129	1.424	0.01794	0.85211	*lgals3bpb*
LOC110536449	36.149	92.257	1.352	0.00903	0.64724	*ccl21*
LOC118944410	86.092	215.650	1.325	0.00003	0.02171	*khdc4*
LOC100136325	12.548	30.690	1.290	0.04838	1.00000	*tapbp (tapasin)*
LOC110487423	76.572	174.198	1.186	0.01325	0.76473	*gys1*
LOC110523157	100.732	226.009	1.166	0.00385	0.46046	
LOC110535266	42.342	89.439	1.079	0.03356	0.98729	*btn1a1*
LOC110488737	23.903	50.072	1.067	0.04936	1.00000	*aipl1*
LOC110533231	849.230	1723.481	1.021	0.00002	0.01895	*banf1*
			**Kidney**			
ccl19a.1	217.748	1501.344	2.786	0.00000	0.00044	*ccl19*
hsp47	346.565	713.408	1.042	0.02781	1.00000	*serpinh1*
lect2	926.671	6569.135	2.826	0.00001	0.01923	*lect2*
LOC100136187	0.296	36.674	6.954	0.00000	0.00045	
LOC100136325	28.663	76.644	1.419	0.02815	1.00000	*tapbp (tapasin)*
LOC110487423	41.075	122.236	1.573	0.00577	1.00000	*gys1*
LOC110488725	21.339	576.671	4.756	0.00000	0.00000	*ffar3*
LOC110488737	0.000	10.006	Inf	0.00496	1.00000	*aipl1*

LOC110495727	49.625	126.489	1.350	0.02732	1.00000	*ifi44l*
LOC110496224	618.390	11632.005	4.233	0.00184	0.69673	*(h2-q9)*
LOC110497998	185.380	876.761	2.242	0.00002	0.02850	
LOC110498757	20.182	59.316	1.555	0.04273	1.00000	*fmnl1*
LOC110507168	18.934	57.298	1.597	0.01810	1.00000	
LOC110509875	591.418	3992.750	2.755	0.00020	0.16403	
LOC110510228	275.360	914.445	1.732	0.00040	0.26609	
LOC110510403	2.618	55.487	4.406	0.00002	0.03049	*mfap4*
LOC110518069	0.296	30.599	6.692	0.02602	1.00000	*gvin1*
LOC110518874	27.943	109.648	1.972	0.00126	0.59625	*ube2d4*
LOC110519256	37.552	138.306	1.881	0.00129	0.59625	*calhm6*
LOC110523157	3273.788	7360.237	1.169	0.01999	1.00000	
LOC110524047	24.150	123.280	2.352	0.00248	0.85915	*lgals3bpb*
LOC110525408	110.593	242.425	1.132	0.03570	1.00000	*gda*
LOC110528445	240.985	1478.582	2.617	0.00449	1.00000	*ifi44*
LOC110528546	548.738	1124.281	1.035	0.04809	1.00000	*rnf213a*
LOC110530481	2.537	106.165	5.387	0.00000	0.00016	
LOC110531529	12.191	83.891	2.783	0.00252	0.85915	*mefv*
LOC110531659	13.425	114.681	3.095	0.01060	1.00000	*gvin1*
LOC110533231	3718.752	10476.705	1.494	0.03067	1.00000	*banf1*
LOC110533271	48.931	238.782	2.287	0.00005	0.05213	
LOC110533568	153.676	330.878	1.106	0.03381	1.00000	*derl2*
LOC110535266	33.709	102.453	1.604	0.01276	1.00000	*btn1a1*
LOC110536048	75.746	1978.947	4.707	0.00000	0.00000	*sds*
LOC110536252	100.255	319.266	1.671	0.00207	0.75392	*s100a1*
LOC110536323	223.688	646.496	1.531	0.00348	1.00000	*ggh*
LOC110536449	274.995	663.221	1.270	0.01332	1.00000	*ccl21*
LOC110537685	96.738	265.734	1.458	0.00820	1.00000	*h2-aa (ha2q)*
LOC110538203	47.554	689.064	3.857	0.00000	0.00000	*c1ql2*
LOC118936255	79.079	1450.103	4.197	0.00004	0.04527	
LOC118936275	0.353	50.706	7.168	0.00000	0.00001	
LOC118941484	0.000	31.860	Inf	0.00044	0.28330	
LOC118944410	89.102	215.395	1.273	0.02804	1.00000	*khdc4*
LOC118964557	854.417	3990.971	2.224	0.00853	1.00000	
LOC118964931	6.541	143.909	4.460	0.00021	0.16452	*saa3*
si:ch211-284e13.9	70.320	211.406	1.588	0.00509	1.00000	
tymp	68.092	236.540	1.797	0.00173	0.68675	*tymp*

Table S3. The DEG lists of Figure 2 (Down-regulated in IT).

Gene id (NCBI)	Base Mean CT	Base Mean IT	log$_2$FC	*p* val	padj	Gene symbol
			**Brain**			
LOC110485559	31.98858	4.92125	-2.700	0.00040	0.13441	*pdgfa*
LOC110522641	383.38639	130.20371	-1.558	0.03667	0.99696	*fos*
LOC110529274	231.64694	105.36848	-1.136	0.02661	0.93851	*fos*

LOC110538343	32.77868	6.14627	-2.415	0.00103	0.22611	*gimap4*
LOC118937699	99.29079	23.54994	-2.076	0.00000	0.00612	
			**Kidney**			
LOC110485559	90.63801	26.82252	-1.757	0.03644	1.00000	*pdgfa*
LOC110522641	569.23223	84.55087	-2.751	0.01721	1.00000	*fos*
LOC110529274	475.64288	86.26071	-2.463	0.01527	1.00000	*fos*
LOC110538343	94.24856	11.68663	-3.012	0.00001	0.01638	*gimap4*
LOC118937699	191.615	59.700	-1.682	0.00202	0.75037	

Table S4. The enriched GO terms and KEGG pathway lists of Figure 2 ~ 4.

Term	Function description		Up- or downregulation
	**The enriched GO terms that are share in comparisons of IB vs. CB and IK vs. CK**		
GO:0045087	innate immune response	biological process	Up
GO:0006955	immune response	biological process	Up
GO:0006953	acute-phase response	biological process	Up
GO:0051607	defense response to virus	biological process	Up
GO:0042742	defense response to bacterium	biological process	Up
GO:0006954	inflammatory response	biological process	Up
	**The enriched GO terms that are specifically identified in IB vs. CB**		
GO:0003796	lysozyme activity	molecular function	Up
GO:0002474	antigen processing and presentation of peptide antigen via MHC class I	biological process	Up
GO:0045824	negative regulation of innate immune response	biological process	Up
GO:0048266	behavioral response to pain	biological process	Up
GO:0002755	MyD88-dependent toll-like receptor signaling pathway	biological process	Up
GO:2000379	positive regulation of reactive oxygen species metabolic process	biological process	Up
GO:0071356	cellular response to tumor necrosis factor	biological process	Up
GO:0006952	defense response	biological process	Up
GO:0032088	negative regulation of NF-kappaB transcription factor activity	biological process	Up
GO:0071346	cellular response to interferon-gamma	biological process	Up
GO:0050728	negative regulation of inflammatory response	biological process	Up
GO:0042612	MHC class I protein complex	cellular component	Up

GO:0071222	cellular response to lipopolysaccharide	Biological process	Up
GO:0099160	postsynaptic intermediate filament cytoskeleton	cellular component	Down
GO:0099182	presynaptic intermediate filament cytoskeleton	cellular component	Down
GO:0099184	structural constituent of postsynaptic intermediate filament cytoskeleton	molecular function	Down
GO:0005344	oxygen carrier activity	molecular function	Down
GO:0014012	peripheral nervous system axon regeneration	biological process	Down
GO:0019825	oxygen binding	molecular function	Down
GO:0019896	axonal transport of mitochondrion	biological process	Down
GO:0098981	cholinergic synapse	cellular component	Down
GO:0060074	synapse maturation	biological process	Down
GO:1904115	axon cytoplasm	cellular component	Down
GO:0021510	spinal cord development	biological process	Down
GO:0048812	neuron projection morphogenesis	biological process	Down
GO:0007218	neuropeptide signaling pathway	biological process	Down
GO:0043524	negative regulation of neuron apoptotic process	biological process	Down
GO:0051301	cell division	biological process	Down
GO:0007059	chromosome segregation	biological process	Down
GO:0000777	condensed chromosome kinetochore	cellular component	Down
GO:0007049	cell cycle	biological process	Down
GO:0006695	cholesterol biosynthetic process	biological process	Down
GO:0046872	metal ion binding	molecular function	Down
	**The enriched GO terms that are specifically identified in IK vs. CK**		
GO:0034987	immunoglobulin receptor binding	molecular function	Up
GO:0048247	lymphocyte chemotaxis	biological process	Up
GO:0034097	response to cytokine	biological process	Up
GO:0003823	antigen binding	molecular function	Up

GO:0002720	positive regulation of cytokine production involved in immune response	biological process	Up
GO:0002879	positive regulation of acute inflammatory response to non-antigenic stimulus	biological process	Up
GO:0090276	regulation of peptide hormone secretion	biological process	Up
GO:0030593	neutrophil chemotaxis	biological process	Up
GO:0071347	cellular response to interleukin-1	biological process	Up
GO:0002385	mucosal immune response	biological process	Up
GO:0014061	regulation of norepinephrine secretion	biological process	Up
GO:0006958	complement activation, classical pathway	biological process	Up
GO:0070266	necroptotic process	biological process	Up
GO:0061844	antimicrobial humoral immune response mediated by antimicrobial peptide	biological process	Up
GO:0001816	cytokine production	biological process	Up
GO:0009617	response to bacterium	biological process	Up
GO:0002250	adaptive immune response	biological process	Up
GO:0005328	neurotransmitter: sodium symporter activity	molecular function	Down
GO:0007269	neurotransmitter secretion	biological process	Down
GO:0048167	regulation of synaptic plasticity	biological process	Down
GO:0017157	regulation of exocytosis	biological process	Down
GO:0008021	synaptic vesicle	cellular component	Down
GO:0042734	presynaptic membrane	cellular component	Down
GO:0007268	chemical synaptic transmission	biological process	Down
GO:0098794	postsynapse	cellular component	Down
GO:0007399	nervous system development	biological process	Down
**The enriched KEGG pathway that are share in comparisons of IB vs. CB and IK vs. CK**			
ko04145	Phagosome		Up
ko04612	Antigen processing and presentation		Up
ko04650	Natural killer cell mediated cytotoxicity		Up
ko04610	Complement and coagulation cascades		Up
ko04917	Prolactin signaling pathway		Up
ko04620	Toll-like receptor signaling pathway		Up

ko04668	TNF signaling pathway	Up
	**The enriched KEGG pathway that are specifically identified in IB vs. CB**	
ko04670	Leukocyte transendothelial migration	Up
ko04664	Fc epsilon RI signaling pathway	Up
ko04666	Fc gamma R-mediated phagocytosis	Up
ko04611	Platelet activation	Up
ko04662	B cell receptor signaling pathway	Up
ko04066	HIF-1 signaling pathway	Up
ko04726	Serotonergic synapse	Up
ko04725	Cholinergic synapse	Up
ko04723	Retrograde endocannabinoid signaling	Up
ko04925	Aldosterone synthesis and secretion	Up
ko04912	GnRH signaling pathway	Up
ko04660	T cell receptor signaling pathway	Up
ko04744	Phototransduction	Up
ko04713	Circadian entrainment	Up
ko04115	p53 signaling pathway	Down
ko04110	Cell cycle	Down
ko03030	DNA replication	Down
ko00100	Steroid biosynthesis	Down
ko04114	Oocyte meiosis	Down
ko04914	Progesterone-mediated oocyte maturation	Down
	**The enriched KEGG pathway that are specifically identified in IK vs. CK**	
ko04621	NOD-like receptor signaling pathway	Up
ko04060	Cytokine-cytokine receptor interaction	Up
ko04064	NF-kappa B signaling pathway	Up
ko04622	RIG-I-like receptor signaling pathway	Up
ko04640	Hematopoietic cell lineage	Up
ko04915	Estrogen signaling pathway	Up
ko04978	Mineral absorption	Up
ko04918	Thyroid hormone synthesis	Up
ko04727	GABAergic synapse	Up
ko04910	Insulin signaling pathway	Up
ko04911	Insulin secretion	Up

Table S5. Up- regulated genes enriched in GO terms and KEGG pathway of Figure 3.

Gene id (NCBI)	Base Mean CT	Base Mean IT	$\log_2$FC	*p* val	Gene symbol
		**in GO terms**			
acod1	0.304638	35.96333	6.883289	0.000902	*acod1*
ifit8	3.534241	17.48511	2.306655	0.018691	*ifit5*
LOC110488725	1.057425	9.913805	3.228883	0.030871	*ffar3*
LOC110489027	1.629379	16.194	3.313065	0.004869	*c3*
LOC110491319	15.14044	67.56694	2.15791	6.34E-05	*unc13d*
LOC110491891	64.63644	386.686	2.580743	0.00016	*b2m*
LOC110492590	2.103307	14.90984	2.825533	0.012482	*cd163*
LOC110495707	4.103801	19.61543	2.256956	0.020349	*c1qa*
LOC110497949	11.49086	51.78458	2.172036	0.000151	*thbs1*
LOC110498119	1.025876	13.35407	3.702351	0.006591	*rsad2*

LOC110505949	4.959402	32.91499	2.730507	0.000931	*trbc2*
LOC110515930	0.304638	10.79707	5.147402	0.005205	*a2m*
LOC110520614	6.876792	36.5034	2.408223	0.000751	*tlr8*
LOC110528067	0.678146	14.19384	4.387526	0.015416	*vwa1*
LOC110537685	2.413717	12.67837	2.393041	0.044989	*h2-aa*
LOC118937842	1.255871	10.24398	3.028016	0.042204	*nlrc3*
nos2	3.08609	20.96712	2.764277	0.003582	*nos2*
si:dkey-195m11.11	0.988555	9.664806	3.289348	0.035716	*cd163*
tlr9	1.051654	9.869169	3.230269	0.031189	*tlr7*
LOC110491319	15.14044	67.56694	2.15791	6.34E-05	*unc13d*
		**in KEGG pathways**			
gngt2a	1.523188	12.41836	3.027308	0.026902	*gngt2*
jak3	15.90028	38.39951	1.272036	0.043242	*jak2*
LOC110485801	5.287424	36.02103	2.768203	0.000204	*pik3r5*
LOC110486935	7.272699	56.242	2.951085	1.30E-05	*gng12*
LOC110493519	34.23848	72.3873	1.080118	0.024575	*kcnq5*
LOC110494246	131.9688	279.1713	1.080954	0.037597	*itpr1*
LOC110495697	161.0023	336.8698	1.06511	0.000277	*pld1*
LOC110495726	449.8679	924.9141	1.039818	4.25E-05	*gng7*
LOC110496638	0.304638	89.26498	8.194857	0.034149	*gngt1*
LOC110497890	28.796	86.73426	1.590734	0.000391	*mapk12*
LOC110502637	37.24137	74.67651	1.003748	0.025202	*alox5*
LOC110503405	61.2139	153.9605	1.330629	0.024313	*pde2a*
LOC110505765	33.56828	100.9605	1.588621	0.010053	*prkd1*
LOC110524068	35.54253	81.87587	1.203892	0.015134	*faah*
LOC110525837	30.57188	64.14236	1.069072	0.02641	*htr7*
LOC110528134	52.63318	111.8913	1.088053	0.006341	*prkcd*
LOC110531171	75.93813	154.1699	1.021624	0.003823	*cacna1d*
LOC110531219	17.04996	60.71047	1.832177	0.00083	*cacna1d*
LOC110537357	6.149781	26.90875	2.129468	0.008356	*pde2a*
LOC110538505	11.97423	40.40776	1.754699	0.005454	*pik3r5*
prkd1	34.04948	193.9495	2.509976	3.34E-05	*prkd1*
ptk2ba	34.92987	73.72918	1.077774	0.014699	*ptk2b*
socs2	26.47722	72.8111	1.459406	0.017787	*socs2*
socs3	33.35303	94.01622	1.495092	0.001735	*socs3*
stat1b	60.39384	148.5998	1.298959	0.01594	*stat1*
stat5	15.44298	37.302	1.272302	0.042098	*stat5b*

Table S6. Down-regulated genes enriched in GO terms and KEGG pathway of Figure 3.

Gene id (NCBI)	Base Mean CT	Base Mean IT	log_2FC	*p* val	Gene symbol
		**in GO terms**			
aurka	96.39547	24.08476	-2.00084	0.031522	*aurka-a*
birc5a	124.5477	35.22157	-1.82217	0.03548	*birc5.2*
LOC100136207	1394.917	226.6463	-2.62166	5.50E-05	*hbb1*
LOC110486354	2023.676	386.8942	-2.38697	0.000196	*hba*
LOC110487060	28.80656	4.494836	-2.68006	0.001569	*cenpx*

LOC110495728	102.2698	10.71938	-3.25409	0.042718	*cartpt*
LOC110496244	64.78847	15.8221	-2.0338	0.01422	*cenpa*
LOC110499168	67.04956	10.2889	-2.70414	0.047532	*hba2*
LOC110499816	206.1175	50.7103	-2.02312	0.014788	*aurkb*
LOC110502544	95.92056	23.20092	-2.04766	0.028349	*kif11*
LOC110505148	120.6956	33.6455	-1.84289	0.044724	*ccna2*
LOC110505614	91.29734	24.21404	-1.91473	0.045665	*tacc3*
LOC110514239	21.28077	2.895006	-2.87791	0.003199	*melk*
LOC110514629	16.88755	2.349169	-2.84574	0.007868	*melk*
LOC110520448	82.39218	23.10382	-1.83438	0.041492	*chaf1b*
LOC110525629	733.1227	220.2491	-1.73492	7.54E-05	*nefl*
LOC110538445	924.5028	279.4327	-1.72618	3.02E-05	*hbb*
mis12	51.37544	14.14109	-1.86119	0.026337	*mis12*
ncaph	136.9341	39.98453	-1.77597	0.037188	*ncaph*
ska1	58.01168	18.32026	-1.6629	0.031179	*ska1*
**in KEGG pathways**					
aurka	96.39547	24.08476	-2.00084	0.031522	*aurka-a*
bub1bb	85.12605	38.72676	-1.13627	0.030659	*bub1b*
cdk2	189.8055	93.95335	-1.01451	0.029891	*cdk2*
fdft1	2166.652	1015.099	-1.09385	4.88E-06	*fdft1*
gtse1	86.84332	38.68154	-1.16677	0.011932	*gtse1*
LOC110485563	411.7358	171.028	-1.26749	0.001097	*sqle*
LOC110486705	38.74127	16.80537	-1.20495	0.043341	*ccne2*
LOC110488948	193.1905	64.09482	-1.59174	0.034886	*ccna2*
LOC110489156	234.3847	69.08128	-1.76251	0.027217	*nsdhl*
LOC110500729	100.6876	20.70276	-2.28199	0.004983	*mad2l1*
LOC110505148	120.6956	33.6455	-1.84289	0.044724	*ccna2*
LOC110506398	440.5589	209.6426	-1.0714	9.98E-05	*dhcr7*
LOC110521563	91.58696	39.6538	-1.20768	0.005399	*cdkn2c*
LOC110528595	203.1449	66.03554	-1.62119	0.034943	*mcm2*
LOC110530465	306.2522	137.6075	-1.15416	0.029667	*mcm4*
LOC110530628	463.0816	173.6491	-1.41509	0.037607	*zmcm6-a*
LOC118938208	54.06651	26.91308	-1.00643	0.045186	*rnaseh2a*
LOC118948701	68.70498	20.94799	-1.7136	0.000667	*plk1*
LOC118951919	94.54292	19.90907	-2.24754	0.031991	*tsc2*
LOC118966114	80.4746	36.17323	-1.15361	0.011257	*bub3*
lss	829.4405	393.2304	-1.07676	0.000282	*lss*
nsdhl	601.223	238.2784	-1.33525	6.92E-07	*nsdhl*
orc5	51.0693	21.50108	-1.24805	0.018207	*orc5*
pttg1	64.12554	20.9738	-1.61231	0.010738	*pttg1*
